# Development of a rat model for prophylactic free gingival graft to mitigate orthodontic-related alveolar bone fenestration/dehiscence

**DOI:** 10.3389/fbioe.2026.1858831

**Published:** 2026-08-27

**Authors:** Zifei Shao, Haokun Huang, Wenlong Du, Chengsi Hu, Lanxin Lu, Xian Cheng, Lingling Zhang, Chengri Li, Yanqin Lu

**Affiliations:** 1 Xiangya Stomatological Hospital and Xiangya School of Stomatology, Central South University, Changsha, China; 2 Hunan Engineering Research Center for Oral Digital Intelligence and Personalized Medicine, Changsha, China

**Keywords:** alveolar bone dehiscence, bone remodeling, free gingival graft, orthodontic tooth movement, rat model

## Abstract

**Background:**

Alveolar bone dehiscence/fenestration is common during orthodontic excessive tooth movement, especially in patients with thin gingiva and alveolar bone, compromising treatment outcomes. Free gingival grafting (FGG) shows preventive potential, but its mechanism remains unclear due to limited animal models.

**Methods:**

Forty 6-week-old male Sprague-Dawley (SD) rats were randomized into 3 groups: FGG + force (FGG + orthodontic force), Sham + force (incision/suture without graft + force) and Blank (no surgical procedure + no force). Right incisors served as internal controls. Orthodontic force (80 ± 1 g) was applied 1 week post-FGG. Rats were euthanized at 4, 8, and 12 weeks post-force application. Micro-CT quantified alveolar bone parameters; histological staining (H&E) and immunohistochemistry (OPN, RUNX2) evaluated bone remodeling.

**Results:**

Micro-CT analysis of the overall data (all time points combined) revealed that in the FGG + force group, the left experimental side had significantly greater alveolar bone height, thickness, bone volume, and bone mineral density compared with the contralateral control side (*p* < 0.05). No significant left-right differences were observed in the Sham + force or Blank groups. Histologically, newly formed epithelial pegs confirmed successful graft integration. Immunohistochemistry showed upregulation of osteogenesis-related proteins RUNX2 and OPN in the FGG + force group, consistent with improved bone morphological parameters.

**Conclusion:**

In this study, we established an excessive orthodontic tooth movement model of maxillary anterior teeth in Sprague-Dawley rats to simulate the changes of alveolar bone resorption and bone defects that frequently occur during orthodontic treatment in patients with thin gingiva and thin alveolar bone. Using micro-CT and histopathological examination, we found that performing FGG prior to orthodontic intervention alleviated orthodontic force-induced alveolar bone resorption during excessive tooth movement. This study provides a referable rat model for exploring the regulatory effect of keratinized gingival grafting on alveolar bone remodeling, and lays a preliminary experimental foundation for the prophylactic application of FGG in orthodontic patients with a thin gingiva-thin bone phenotype.

## 1 Introduction

Malocclusion, a complex dentomaxillofacial deformity arising from multifactorial growth disturbances, is corrected through orthodontic tooth repositioning—a process that relies on the balanced resorption and formation of alveolar bone. Disruption of this delicate balance, often triggered by excessive orthodontic force, large-scale tooth movement, or preexisting thin gingiva/bone phenotypes, can lead to alveolar bone fenestration or dehiscence (Yagci et al., 2012; Nalbantoğlu and Yanık, 2023). These defects not only compromise treatment efficacy and long-term stability but also pose clinical challenges: fenestration manifests as localized root-surface bone defects with intact alveolar ridges (exposing roots to the gingiva or periosteum), while dehiscence presents as “V”-shaped defects extending from the root surface to the alveolar ridge (de Resende et al., 2019). Epidemiologically, the incidence of these defects in the general population’s natural dentition is approximately 8%, but this rate rises sharply to 23% in patients with bilateral protrusion or skeletal Class III malocclusion, and reaches 33% in those undergoing camouflage orthodontic treatment (Yagci et al., 2012; Enhos et al., 2012; Evangelista et al., 2010). A critical risk factor is labial-buccal alveolar bone thickness < 2 mm, as tooth movement in such cases significantly increases defect likelihood; even minor movement toward the thin bone/gingiva side can breach cortical bone and cause irreversible resorption (Allais and Melsen, 2003; Baysal et al., 2013). While cone beam computer tomography (CBCT) enables *in vivo* measurement of alveolar bone changes (Evangelista et al., 2010; Sun et al., 2022; Dominiak et al., 2021), ethical constraints and sampling difficulties prevent histological analysis of dehiscence progression—leaving clinicians without proven preventive strategies and patients uncertain about balancing complication risks with treatment needs.

Current strategies to mitigate orthodontic-induced alveolar bone defects vary in efficacy and practicality. Cortical osteotomy, a common intervention, fails to reduce post-treatment defect risk when performed without concurrent grafting (Brugnami et al., 2018). Bone grafting combined with barrier membranes enhances osteogenesis but is limited by uneven membrane degradation and procedural complexity, which increase clinical burden. In contrast, free gingival grafting (FGG) offers distinct advantages: it is cost-effective and technically simple. First reported by Nabers (1966), FGG involves transplanting autologous keratinized gingiva to widen attached soft tissue and cover root recessions. Since the mid-20th century, it has been used to address inadequate attached gingiva in orthodontics (Girardot, 2013; Chambrone and Chambrone, 2005). Postoperatively, patients show significant soft tissue thickening, and a well-executed FGG may also slightly increase bone volume at the surgical site (Consensus report, 1996). Consequently, FGG is increasingly used prophylactically before orthodontics—such as with pre-treatment keratinized width <2 mm (Dorfman, 1978; Scheyer et al., 2015),planned labial movement in thin bone/gingiva (Wennström et al., 1987), or alveolar bone fractures requiring further tooth movement (Consensus report, 1996). The 2015 guidelines from the American Academy of Periodontology (AAP) further endorse FGG as a preventive measure: it is recommended for patients requiring labial tooth movement or arch expansion with preexisting recession or thin phenotypes, with the goal of achieving ≥ 2 mm keratinized gingiva to reduce fenestration/dehiscence risk (Consensus report, 1996; Kim and Neiva, 2015). Despite these benefits, FGG’s clinical adoption remains limited due to an unclear underlying mechanism of action. Regarding the possible mechanisms by which soft tissue grafting promotes hard tissue augmentation, the following are currently recognized. First, the barrier effect of the grafted tissue (Kan et al., 2009; Shirakata et al., 2019). Second, surgical trauma leading to the release of osteoprogenitor cells from the periosteum-alveolar bone interface, which then become activated, proliferate, and form new bone (Pack et al., 1991; Paolantonio et al., 2019). Third, proliferation and differentiation of mesenchymal stem cells derived from the grafted tissue, periosteum, or periodontal ligament (Cairo, 2000; Xu et al., 2020).

While FGG exerts a protective effect on local alveolar bone, critical knowledge gaps hinder its standardized application—particularly regarding its mechanism of promoting bone health. Existing research on FGG is primarily clinical and human-focused: studies have demonstrated that FGG reduces inflammation via barrier effects, maintains marginal bone height (Tavelli et al., 2021; Kungsadalpipob et al., 2020; Thoma et al., 2018), and can induce alveolar bone augmentation (Chambrone and Chambrone, 2005; Karaca et al., 2015; Keceli and Hendek, 2014; Pasquinelli, 1995). Our research group has also contributed evidence, showing increased alveolar bone height and thickness, as well as reduced defect incidence, in adult orthodontic patients following FGG (Zhang et al., 2023). However, the specific biological pathways driving these effects remain unelucidated; potential mechanisms (e.g., physical barrier functions, contributions from periosteal progenitor cells, or actions of graft-derived stem cells) have not been validated. A major limitation to addressing this gap is the lack of appropriate animal models.

To address the aforementioned research gaps and validate the effect that FGG exerts a protective effect on local alveolar bone, this study developed a standardized animal model using Sprague-Dawley (SD) rats. First, the model was designed to simulate clinical conditions by inducing excessive tooth movement of anterior teeth in rats, thereby recapitulating the scenario of orthodontic treatment that risks alveolar bone dehiscence. Second, preventive FGG was incorporated into the model to replicate the clinical practice of pre-orthodontic gingival grafting for thin phenotypes. Third, the model was used to comprehensively characterize FGG-promoted alveolar bone remodeling during orthodontic tooth movement—enabling histological and molecular analyses that are not feasible in human studies, and directly addressing the knowledge gap in soft tissue-to-bone transition. Finally, the study aimed to confirm FGG’s ability to promote alveolar bone augmentation under orthodontic force, with the broader goal of providing a theoretical basis for the clinical application of FGG. If validated, this model could transform treatment strategies for patients with a thin gingival/bone phenotype, and strengthen the status of FGG as an alternative to complicated bone grafting procedures. Future research based on this model will likely culminate in the development of new, safe and favorable treatment modalities.

## 2 Materials and methods

### 2.1 Animals and groups

Sample size was calculated using PASS 15.0 software (NCSS, LLC, Kaysville, UT, United States) for a two-sided Wilcoxon signed-rank test (paired design) with α = 0.05 and power = 80%. Based on a probability of superiority of 0.80 (derived from pilot data), the minimum required sample size was 8 rats for each group (Young et al., 2013). Given the higher surgical complexity and anticipated postoperative risks in the FGG + force group, an additional 4 rats were added to this group to compensate for potential losses. Forty healthy male 6-week-old Sprague-Dawley rats (200–250 g; Hunan SJA Laboratory Animal Co., Ltd.) were acclimated for 1 week (12 h light/dark cycle, 22 °C–25 °C, 55%–60% humidity; 3 rats/cage) and divided into three groups according to a random number method (FGG + force group, n = 16; Sham + force group, n = 12; Blank group, n = 12, the experimental unit is a single animal):

1. FGG + force group: FGG at left incisor followed by orthodontic appliance 1 week post-healing. And the right incisor served as the control group.
2. Sham + force group: Gingival incision and suturing (no graft) on left incisor, with appliance 1 week later. And the right incisor served as the control group.
3. Blank group: No orthodontic appliance was placed, and no treatment was performed on the gingiva or palate.

In addition, we adopted a within-subject control design. The side that received the orthodontic appliance without any gingival treatment was designated as the Force group. Right incisors served as internal controls. V-shaped appliances applied excessive force to incisors 1 week post-grafting. Four rats/group were euthanized at 4, 8, and 12 weeks post-force application. After processing, all the animals must meet the following conditions: there should be no redness or swelling in the observation area, no necrosis occurs, and the tissues should be able to fuse well; otherwise, the animal will be excluded. The design of the experiment, the grouping of the animals, the collection of data and the analysis were all carried out by different experimenters to eliminate any subjective interference. At the end of the experiment, rats were euthanized via intraperitoneal (i.p.) injection of an overdose of sodium pentobarbital (150 mg/kg, 50 mg/mL stock solution in sterile 0.9% NaCl), administered using 2-mL disposable syringes with 26-gauge needles. Euthanasia was confirmed by the permanent loss of righting reflex, cessation of respiratory and cardiac activity, and absence of corneal/pain reflexes (monitored continuously for 5 min). Protocols were approved by Central South University’s Animal Ethics Committee (No.CSU-2022-0001-0075).

### 2.2 Operative procedures

#### 2.2.1 FGG + force group procedures

Sodium pentobarbital (40 mg/kg, i. p.) was loaded in 2-mL disposable syringes to anesthetize rats for 30–60 min.

Recipient site preparation: An incision was made on the gingiva distal to the incisor using a scalpel. The gingiva was bluntly dissected to expose a 4 × 4 mm area of periosteum (the underlying alveolar bone was not exposed).

Donor site preparation: Palatal mucosa (4 × 4 mm) collected between first-third molars via longitudinal incisions with transverse extensions.

Transplant fixation: Graft secured to periosteum with tension-free interrupted 7-0 sutures at incision corners. Buccal muscles were gently pulled to confirm that their movement did not affect graft fixation.

#### 2.2.2 Sham + force operation group procedure

The Sham + force group received identical surgical procedures, including surgical management of the donor site, as the FGG + force group, except that no donor tissue was implanted and only *in-situ* suturing was performed after surgical preparation at the recipient site.

#### 2.2.3 Installation of the force-applying device

A 1-mm hole was drilled between maxillary central incisors. The V-shaped appliance was inserted parallel to force direction (avoiding mucosal compression) and bonded to incisors with dental resin. Teeth were trimmed weekly; powdered diet was provided. Dislodged appliances were rebonded immediately.

### 2.3 Micro-computed tomography (micro-CT)

Specimens were collected at 4, 8, and 12 weeks post-intervention. Following anesthesia, rats underwent cardiac perfusion with saline followed by 4% paraformaldehyde. Maxillae were dissected, fixed in 4% paraformaldehyde for 24 h, and scanned after appliance removal using a micro-CT system (Skyscan 1172, Bruker) at 100 kV, 200 μA, with 10-μm slice thickness (7 min acquisition).

Data were reconstructed in Data Viewer (Bruker). The coronal plane was aligned parallel to the mid-sagittal suture using anatomical landmarks: Bu (lingual palatal incisor apex) and Pr (anterior-inferior alveolar process point). Three measurement planes perpendicular to the Bu-Pr axis were defined at 0 mm (basal), 1 mm (mid), and 2 mm (apical). The buccal bone wall of the tooth corresponding to this 2-mm segment was set as the region of interest (ROI). Alveolar bone thickness was measured at the cervical, middle, and apical root thirds on each plane using CTAn (Bruker). Bone volume (BV) and bone mineral density (BMD) were calculated within the ROI(Grayscale Indexes: 200-255).

### 2.4 Histological staining

Post-micro-CT specimens were decalcified, dehydrated, and embedded in optimal cutting orientation. Serial 4-μm sections were cut parallel to the bone height plane. Sections underwent:

Observation of tissue morphology and structure by H&E staining (G1120, Solarbio, Beijing, China); IHC with primary antibodies: anti-OPN (PSH09-23, Huabio, Hangzhou, China) and anti-RUNX2 (bs-1134R, Bioss, Beijing, China) incubated overnight at 4 °C.

### 2.5 Statistical analysis

All statistical analyses were performed using SPSS software (version 26.0, IBM Corp., Armonk, NY, United States). Data were tested for normality using the Shapiro–Wilk test. Since the data were not normally distributed, they are presented as median with interquartile range (IQR). Intra-group comparisons (between the left experimental side and the right control side within the same rat) were performed using the paired Wilcoxon signed-rank test. A two-sided p < 0.05 was considered statistically significant.

## 3 Results

### 3.1 Construction of the rat model, device setup, and surgical procedures

**FIGURE 1:**
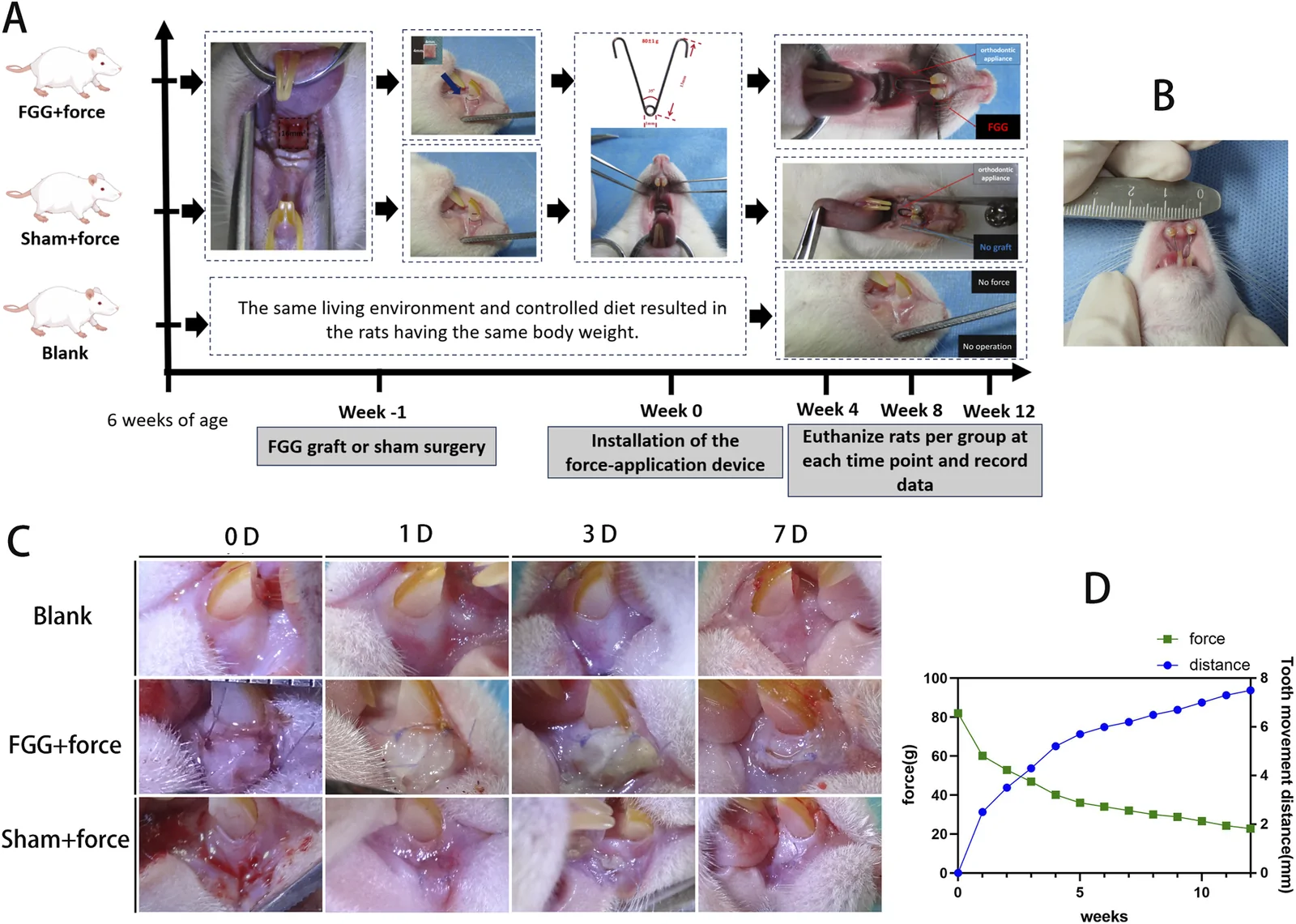
**(A)** Schematic timeline for the experimental design. **(B)** Significant tooth movement was observed 2 weeks post-installation of the force application device. **(C)** Comparison of blank, FGG + force, and sham + force groups' 0-7 days post-treatment healing. **(D)** The relationship between the force values provided by the force device and the distance of tooth movement from week 1 to week 12.

Under anesthesia, a full-thickness keratinized gingival graft measuring 4 × 4 mm (16 mm^2^) with a thickness of 0.5–0.7 mm was collected from the hard palate of rats via scalpel incision combined with blunt dissection (Figure 1A). Hemostasis was achieved by sterile cotton compression; the donor site was sutured and healed completely within 10 days (Supplementary Figure S1A). At the recipient site, an identical incision was made. Submucosal connective tissue was excised (periosteum preserved) to ensure graft-bone adaptation. The graft was secured with tension-free sutures. Successful transplantation required: absence of redness/edema/necrosis, and tissue integration (Figure 1C). No animals were excluded during the experiment. A 0.016-inch Australian wire loop (arm length: 13 mm, loop diameter: 1 mm, arm angle: 35°) delivered 80 ± 1 g initial force (dynamometer-calibrated, Figure 1A). For implantation, a 1-mm cooling-irrigated hole drilled between incisors, loop positioned to avoid gingival contact, etched tooth surfaces bonded with light-cure resin (Powdered diet minimized detachment risk). After the force application device is placed for 2 weeks, 3 mm movement distance (Linear displacement between bilateral incisor medial points) can be observed (Figure 1B). The movement distance increased significantly in the first four weeks, and then the growth rate gradually slowed down. There was no statistically significant difference in the distance of tooth movement among the different groups of rats (triplicate measurements; mean reported; Figure 1D).

### 3.2 Changes in the alveolar bone of rat anterior teeth

**FIGURE 2:**
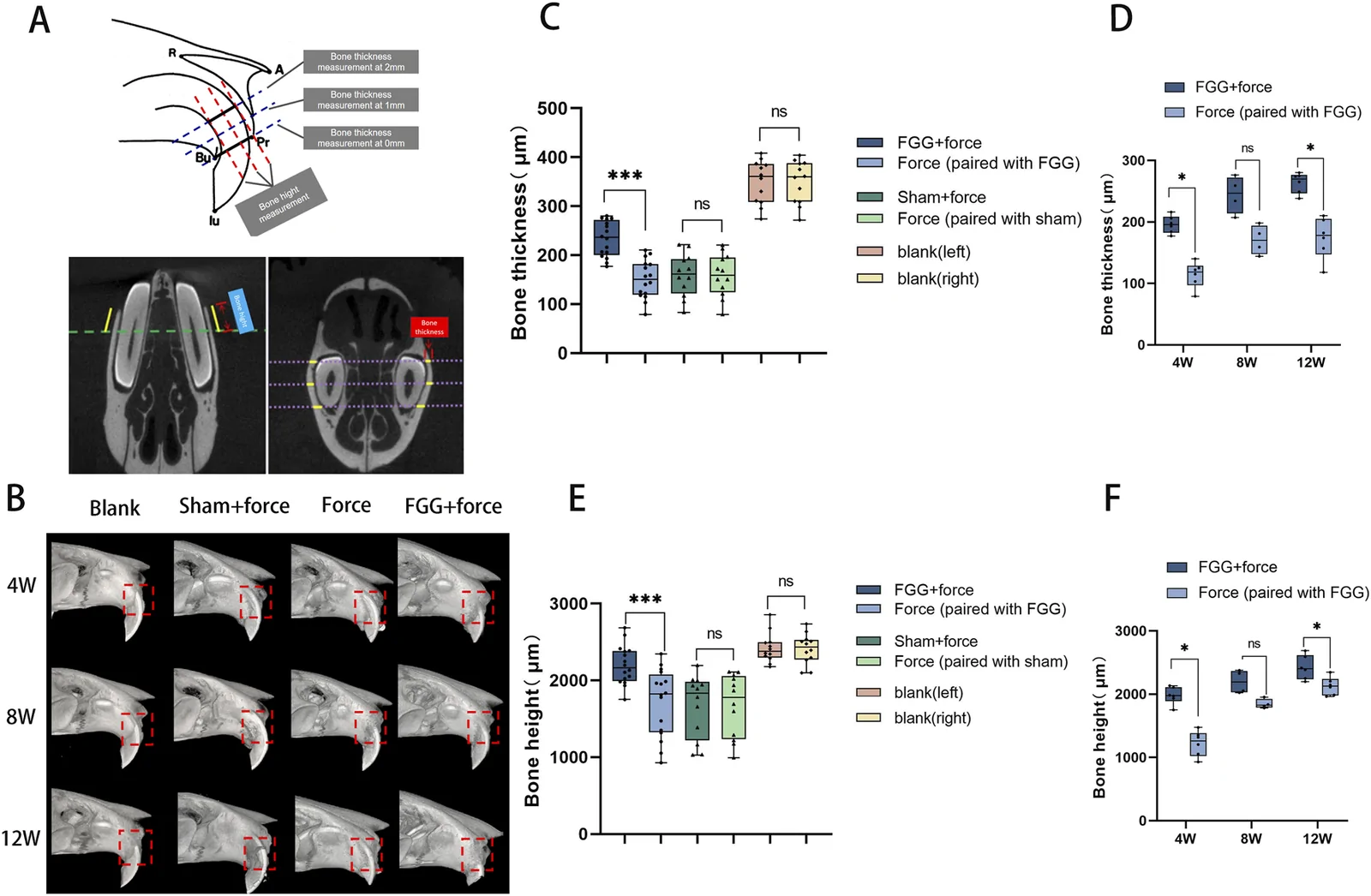
**(A)** Schematic of buccal bone measurement sites in rat anterior teeth, showing bone height and thickness. **(B)** CTvox reconstruction of alveolar bone: The FGG + force group exhibited greater bone volume and less resorption than the Sham + force and Force groups, and was closer to the Blank group. **(C)** Pairwise comparisons of bone thickness among FGG + force, Sham + force and Blank groups. **(D)** Pairwise comparisons of bone thickness in the FGG + force group at 4, 8 and 12 weeks. **(E)** Pairwise comparisons of bone height among FGG + force, Sham + force and Blank groups. **(F)** Pairwise comparisons of bone height in the FGG + force group at 4, 8 and 12 weeks. (*p < 0.05, **p < 0.01, ***p < 0.001).

Alveolar bone thickness was measured at cervical, middle, and apical root thirds on planes 0 mm (basal), 1 mm (mid), and 2 mm (apical) from the Bu-Pr reference plane (Kiliaridis et al., 1985). Bone height was measured perpendicularly from a plane 2 mm apical to the reference plane at cervical, middle, and apical root levels. Figure 2A provides a direct visualization of the measurement planes for bone height and bone thickness, as well as the corresponding morphology within each measurement plane. Micro-CT reconstructions (Figure 2B) revealed that Blank group had intact buccal bone at all time points; Sham + force group and Force group showed progressive fenestration/dehiscence with root exposure (maximal at alveolar crest). The FGG + force group exhibited markedly reduced bone defects during tooth movement compared with the Sham + force operation and Force groups. These findings suggest that FGG exerts a protective effect on local alveolar bone.

Micro-CT (Table 1; Figure 2E) showed that the left side (experimental) had significantly greater alveolar bone height than the right side (control) in the FGG + force group (*p* = 0.001). No significant differences were observed in the Sham + force group or the Blank group. When analyzed by time point (Table 2; Figure 2F), the FGG + force group exhibited significantly higher left-side bone height at 4 weeks (*p* = 0.028) and 12 weeks (*p* = 0.028), whereas the difference at 8 weeks did not reach statistical significance.

**TABLE 1:** Comparison of alveolar bone height between left and right sides in each group.

Group	*n*	Left median (IQR) (µm)	Right median (IQR) (µm)	*P* *
Operation	16	2,166.50 (1993.75–2,384.00)	1825.50 (1,326.75–2079.25)	**0.001**
Sham	12	1836.50 (1,219.00–1984.25)	1778.50 (1,236.25–2055.00)	0.875
Blank	12	2,381.50 (2,295.50–2,497.25)	2,435.00 (2,274.50–2,527.75)	0.754

* Wilcoxon signed-rank test (two-tailed). Significant p-values (<0.05) are bolded.

**TABLE 2:** Comparison of alveolar bone height between left and right sides in the operation group at each time point.

Time	*n*	Left median (IQR) (µm)	Right median (IQR) (µm)	*P* *
4W	6	1977.50 (1888.50–2,112.25)	1,261.50 (1,022.50–1,380.75)	**0.028**
8W	4	2,191.50 (2030.75–2,363.50)	1825.50 (1788.50–1924.75)	0.068
12W	6	2,400.00 (2,235.75–2,614.25)	2,128.00 (1981.00–2,239.75)	**0.028**

* Wilcoxon signed-rank test (two-tailed). Significant p-values (<0.05) are bolded.

As shown in Table 3 and Figure 2C, the FGG + force group had significantly greater bone thickness on the left side compared with the right side (*p* < 0.001). No significant differences were found in the Sham + force Blank groups (Figure 2D). Time-point analysis (Table 4) revealed significant left-right differences at 4 weeks (*p* = 0.028) and 12 weeks (*p* = 0.028), but not at 8 weeks.

**TABLE 3:** Comparison of alveolar bone thickness between left and right sides in each group.

Group	*n*	Left median (IQR) (µm)	Right median (IQR) (µm)	*P* *
Operation	16	236.00 (200.00–271.75)	150.50 (119.00–181.75)	**0.000**
Sham	12	161.50 (121.75–192.00)	159.00 (124.25–194.75)	0.406
Blank	12	360.50 (308.75–386.25)	360.00 (309.25–387.75)	0.812

* Wilcoxon signed-rank test (two-tailed). Significant p-values (<0.05) are bolded.

**TABLE 4:** Comparison of alveolar bone thickness between left and right sides in the operation group at each time point.

Time	*n*	Left median (IQR) (µm)	Right median (IQR) (µm)	*P* *
4W	6	195.50 (182.25–208.50)	118.50 (97.00–128.75)	**0.028**
8W	4	246.50 (213.75–271.75)	170.00 (147.75–193.75)	0.066
12W	6	269.50 (246.25–276.25)	178.00 (147.25–204.75)	**0.028**

* Wilcoxon signed-rank test (two-tailed). Significant p-values (<0.05) are bolded.

**FIGURE 3:**
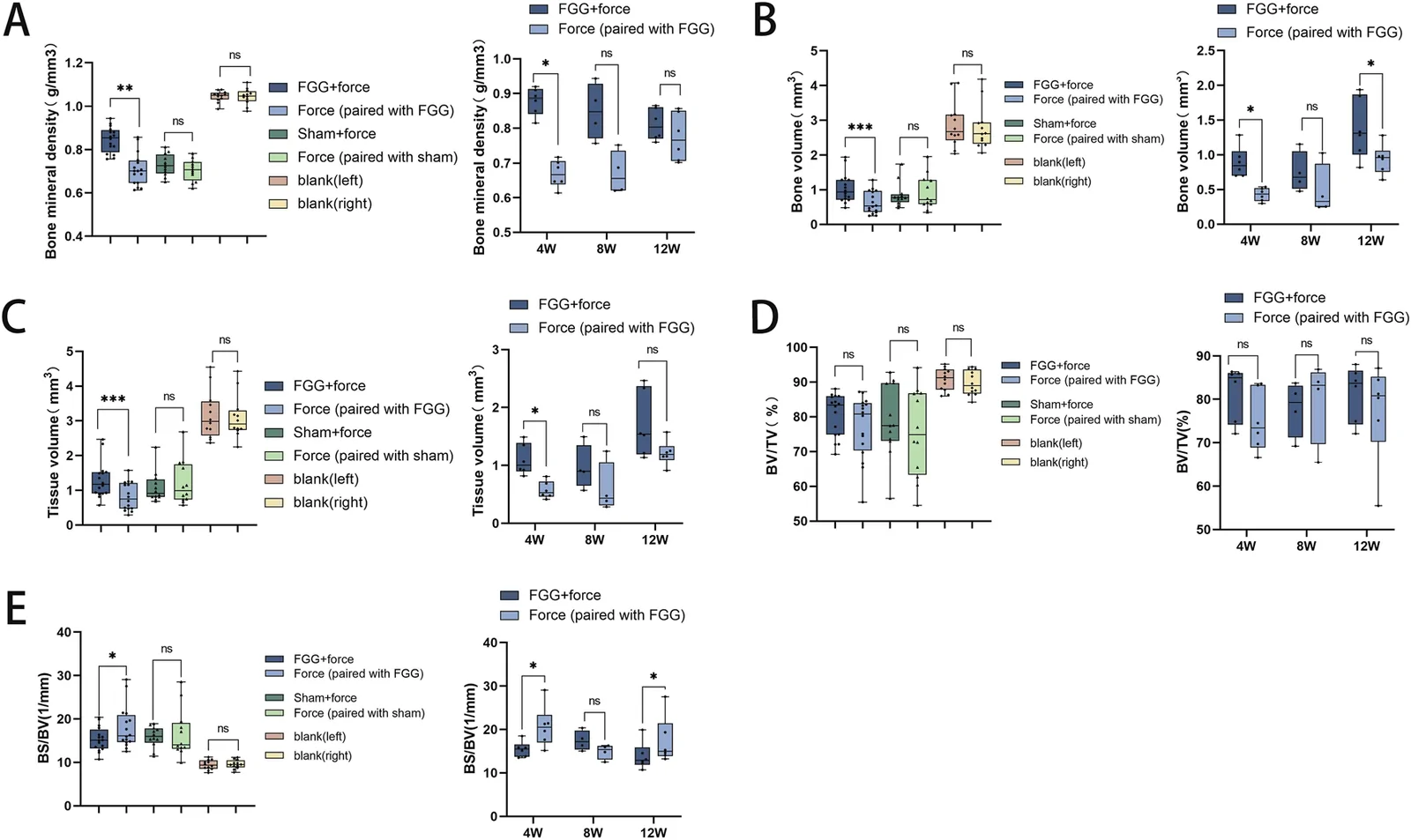
ROI bone tissue analysis by CTan software. **(A)** Pairwise comparisons of bone mineral density (BMD) among FGG + force, Sham + force and Blank groups, and within the FGG + force group at 4, 8 and 12 weeks. **(B)** Pairwise comparisons of bone volume (BV) among FGG + force, Sham + force and Blank groups, and within the FGG + force group at 4, 8 and 12 weeks. **(C)** Pairwise comparisons of tissue volume (TV) among FGG + force, Sham + force and Blank groups, and within the FGG + force group at 4, 8 and 12 weeks. **(D)** Pairwise comparisons of BV/TV among FGG + force, Sham + force and Blank groups, and within the FGG + force group at 4, 8 and 12 weeks. **(E)** Pairwise comparisons of BS/BV among FGG + force, Sham + force and Blank groups, and within the FGG + force group at 4, 8 and 12 weeks. (*p < 0.05, **p < 0.01, ***p < 0.001).

BMD was significantly higher on the left side in the FGG + force group (*p* = 0.002; Table 5; Figure 3A), whereas no differences were observed in the Sham + force or Blank groups. In the FGG + force group (Table 6; Figure 3A), a significant difference was present only at 4 weeks (*p* = 0.028); at 8 weeks and 12 weeks the differences were not statistically significant.

**TABLE 5:** Comparison of alveolar bone mineral density between left and right sides in each group.

Group	*n*	Left median (IQR) (g/mm^3^)	Right median (IQR) (g/mm^3^)	*P* *
Operation	16	0.8545850 (0.7879150–0.8897725)	0.7016950 (0.6468300–0.7488950)	**0.002**
Sham	12	0.7260200 (0.6926050–0.7769300)	0.7067300 (0.6585700–0.7424850)	0.433
Blank	12	1.0507300 (1.0312925–1.0615300)	1.0472,700 (1.0236600–1.0682700)	0.638

* Wilcoxon signed-rank test (two-tailed). Significant p-values (<0.05) are bolded.

**TABLE 6:** Comparison of alveolar bone mineral density between left and right sides in the operation group at each time point.

Time	*n*	Left median (IQR) (g/mm^3^)	Right median (IQR) (g/mm^3^)	*P* *
4W	6	0.8863650 (0.8411700–0.9125400)	0.6670000 (0.6385850–0.7054400)	**0.028**
8W	4	0.8475550 (0.7710400–0.9276475)	0.6550400 (0.6211900–0.7356450)	0.068
12W	6	0.8030650 (0.7706650–0.8607575)	0.7661300 (0.7061150–0.8502250)	0.463

* Wilcoxon signed-rank test (two-tailed). Significant p-values (<0.05) are bolded.

Bone volume (BV) was significantly greater on the left side in the FGG + force group (*p* < 0.001; Table 7; Figure 3B), while no differences were seen in the Sham + force or Blank groups. Time-point analysis (Table 8; Figure 3B) showed significant differences at 4 weeks (*p* = 0.028) and 12 weeks (*p* = 0.028), but not at 8 weeks. Tissue volume (TV) followed a similar pattern (Table 9; Figure 3C): left side significantly higher in the FGG + force group (*p* = 0.001), with no significant differences in the other groups. At individual time points (Table 10; Figure 3C), significance was reached only at 4 weeks (*p* = 0.028).

**TABLE 7:** Comparison of alveolar bone volume between left and right sides in each group.

Group	*n*	Left median (IQR) (mm^3^)	Right median (IQR) (mm^3^)	*P* *
Operation	16	0.9357950 (0.7105650–1.2849850)	0.5260950 (0.3581050–0.9681975)	**0.000**
Sham	12	0.7690800 (0.6416475–0.8702850)	0.7106650 (0.5818650–1.2863775)	0.875
Blank	12	2.6784900 (2.4275650–3.1651550)	2.6151600 (2.3258250–2.9389775)	0.272

* Wilcoxon signed-rank test (two-tailed). Significant p-values (<0.05) are bolded.

**TABLE 8:** Comparison of alveolar bone volume between left and right sides in the operation group at each time point.

Time	*n*	Left median (IQR) (mm^3^)	Right median (IQR) (mm^3^)	*P* *
4W	6	0.8438350 (0.7013100–1.0519650)	0.4334650 (0.3335275–0.5196375)	**0.028**
8W	4	0.6772000 (0.5110400–1.0492950)	0.3254900 (0.2489000–0.8688200)	0.068
12W	6	1.3098950 (1.0040900–1.8694775)	0.9587150 (0.7556400–1.0570575)	**0.028**

* Wilcoxon signed-rank test (two-tailed). Significant p-values (<0.05) are bolded.

**TABLE 9:** Comparison of alveolar tissue volume between left and right sides in each group.

Group	*n*	Left median (IQR) (mm^3^)	Right median (IQR) (mm^3^)	*P* *
Operation	16	1.172970 (0.9113150–1.5167650)	0.7490350 (0.4748750–1.2073300)	**0.001**
Sham	12	0.9143500 (0.8075275–1.3097475)	0.9885650 (0.7339800–1.7472000)	0.695
Blank	12	2.9853800 (2.5806725–3.5542525)	2.9048100 (2.7350275–3.2930275)	0.388

* Wilcoxon signed-rank test (two-tailed). Significant p-values (<0.05) are bolded.

**TABLE 10:** Comparison of alveolar tissue volume between left and right sides in the operation group at each time point.

Time	*n*	Left median (IQR) (mm^3^)	Right median (IQR) (mm^3^)	*P* *
4W	6	1.0020450 (0.8954575–1.3856200)	0.5285350 (0.4613375–0.7188225)	**0.028**
8W	4	0.8989000 (0.6489000–1.3477850)	0.4300300 (0.3101500–1.0521700)	0.068
12W	6	1.5379450 (1.1918300–2.3677800)	1.1906900 (1.0930450–1.3317725)	0.116

* Wilcoxon signed-rank test (two-tailed). Significant p-values (<0.05) are bolded.

BV/TV did not differ significantly between left and right sides in any group (Table 11; Figure 3D). However, bone surface (BS)/BV was significantly lower on the left side in the FGG + force group (*p* = 0.044; Table 12; Figure 3E), indicating a more compact bone structure. Time-point analysis (Table 13; Figure 3E) revealed significantly lower BS/BV on the left side at 4 weeks (*p* = 0.046) and 12 weeks (*p* = 0.028), but not at 8 weeks.

**TABLE 11:** Comparison of alveolar BV/TV between left and right sides in each group.

Group	*n*	Left median (IQR) (%)	Right median (IQR) (%)	*P* *
Operation	16	83.3724250 (74.8796850–85.8895350)	80.7876500 (70.3461175–83.901050)	0.255
Sham	12	77.4510350 (73.0921750–89.6743550)	74.8917750 (63.3507225–86.7221850)	0.530
Blank	12	91.1807700 (87.8302700–93.5723400)	88.9323600 (86.6837475–93.5707950)	0.209

* Wilcoxon signed-rank test (two-tailed). Significant p-values (<0.05) are bolded.

**TABLE 12:** Comparison of alveolar BS/BV between left and right sides in each group.

Group	*n*	Left median (IQR) (1/mm)	Right median (IQR) (1/mm)	*P* *
Operation	16	15.1079100 (13.2164100–17.5335025)	16.1167650 (14.6773375–20.8823200)	**0.044**
Sham	12	15.9401450 (14.5008400–17.7802875)	14.0294000 (13.1116975–19.0731425)	1.000
Blank	12	9.3654450 (8.5438150–10.4665750)	9.4841500 (8.8437000–10.4497575)	0.695

* Wilcoxon signed-rank test (two-tailed). Significant p-values (<0.05) are bolded.

**TABLE 13:** Comparison of alveolar BS/BV between left and right sides in the operation group at each time point.

Time	*n*	Left median (IQR) (1/mm)	Right median (IQR) (1/mm)	*P* *
4W	6	15.4715700 (13.7266725–16.5166250)	20.4767600 (17.0167475–23.3440025)	**0.046**
8W	4	17.1427150 (15.3854575–19.733525)	15.3785150 (13.0900300–16.2072525)	0.144
12W	6	12.9184800 (11.8860900–15.8776250)	14.9599350 (13.8493625–21.4071175)	**0.028**

* Wilcoxon signed-rank test (two-tailed). Significant p-values (<0.05) are bolded.

### 3.3 Histological manifestations of the animal model

**FIGURE 4:**
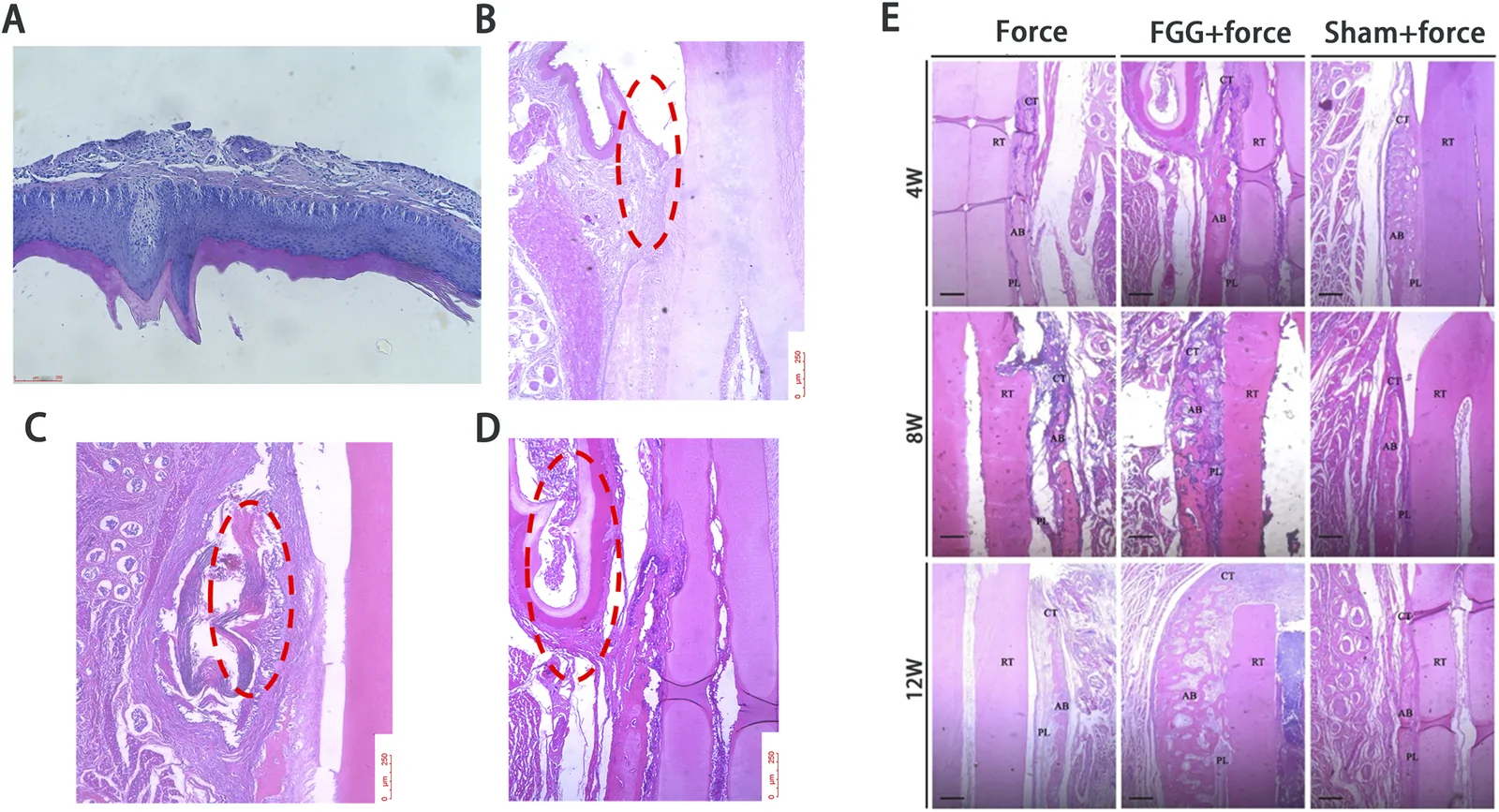
Results of H&E staining after different treatments. **(A)** Keratinized gingival mucosa in the upper palate of rats. **(B)** The gingiva in the anterior teeth area of rats showed no epithelial spike (red circle). **(C)** 4 weeks after keratinized gingival transplantation surgery, epithelial spike processes (red circles) were found in the surgical recipient area. **(D)** 8 weeks after keratinized gingival transplantation surgery, the epithelial spike process still persists (red circle). **(E)** Tissue sections in the force group, FGG + force group and sham + force group at different times. (RT: root, CT: connective tissue, AB: alveolar bone, PL: periodontal ligament) (Black scale length 250 μm).

Histological assessment of donor palatal mucosa via H&E and Masson staining revealed characteristic features including regularly bundled collagen fibers, sparse vascular distribution, and prominent epithelial pegs (Figure 4A; Supplementary Figure S1B). Notably, these epithelial pegs were entirely absent in the native alveolar mucosa of SD rats (Figure 4B). Following free gingival transplantation, recipient sites developed newly formed epithelial pegs by 4 weeks post-operation, which persisted through the 8-week observation period (Figures 4C,D).

H&E staining results of the Force group, FGG + force group, and Sham + force group at different time points are shown in Figure 4E. At 4 weeks, the Force group exhibited a decrease in alveolar crest height and discontinuity of the surface cortical bone; FGG + force group showed that the transplanted palatal mucosal tissue completely covered the alveolar bone surface, with stable attachment between the subepithelial connective tissue and the bone surface, and discontinuity of the alveolar cortical bone with reduced thickness after force application; Sham + force group showed a decrease in alveolar bone height, discontinuity of cortical bone, and penetrating defects in the internal and external bone plates.

At 8 weeks, bone resorption reached maximal severity across Force group. Force group showed a further decrease in alveolar bone height, complete destruction of cortical bone continuity, connective tissue surrounding the resorbed alveolar bone, and scattered bone resorption lacunae on the bone surface. FGG + force group demonstrated increased alveolar thickness relative to 4-week baselines, with histological evidence of active remodeling including numerous Haversian canals within bone matrices. Sham + force group showed slight recovery of cortical bone continuity compared with 4 weeks, but the alveolar crest height was still lower than that of FGG + force group.

At 12 weeks, FGG + force group exhibited substantial gains in both vertical bone height and buccolingual thickness. Histological sections revealed osseous integration between newly formed cementum and alveolar bone, surrounded by dense connective tissue organization. In contrast, Force and Sham + force group maintained thin alveolar structures with persistent cortical discontinuity. Sham + force group specifically showed ongoing destruction at the alveolar crest region without evidence of late-stage recovery.

### 3.4 Detection of the expression of osteogenesis-related proteins in tissues

Immunohistochemical labeling of osteogenesis-related proteins OPN and RUNX2 showed that in Blank group without force application or treatment, the buccal bone walls of the bilateral anterior teeth were thin and uniform, with inactive osteogenic and osteoclastic activities in the alveolar bone (Supplementary Figures S2A,B). Quantitative analysis of positive expression using ImageJ software also showed no significant difference in the expression of OPN (Supplementary Figure S2C) and RUNX2 (Supplementary Figure S2D) between the left and right sides of the anterior teeth in Blank group.

**FIGURE 5:**
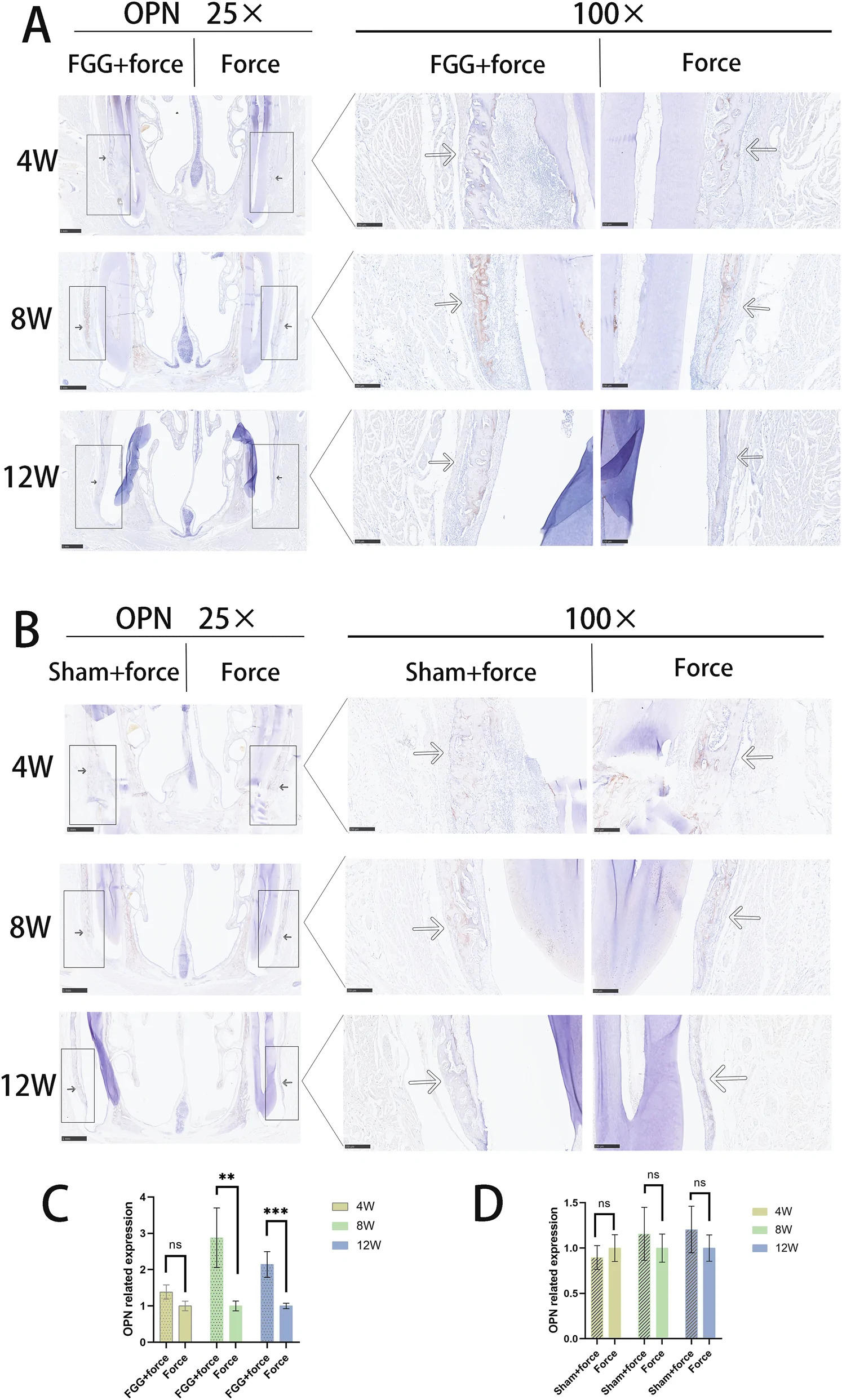
OPN immunohistochemical staining. **(A)** The positive expression of OPN in the bone tissue of the FGG + force group was slightly higher than force group at 4 weeks and 12 weeks, and significantly higher at 8 weeks. **(B)** Compared with the FGG + force group, the expression distribution of OPN in the sham + force operation group was more uniform, and the expression differences of bilateral OPN at 4 weeks, 8 weeks and 12 weeks were smaller. **(C,D)** The positive degree of immunohistochemical results of OPN and RUNX2 was analyzed by ImageJ software and represented by bar charts. (The arrow points to the location for observation and analysis.) (*p < 0.05, **p < 0.01, ***p < 0.001).

Immunohistochemical staining of OPN in FGG + force group (Figure 5A) and Sham + force group (Figure 5B) with device force application showed that at 4 weeks, both FGG + force and Sham + force group demonstrated active bone remodeling with prominent resorption lacunae. OPN expression appeared as scattered foci near lacunar margins without significant side-to-side variation. By 8 weeks, OPN staining in FGG + force group was significantly enhanced, widely distributed on the bone tissue surface, and the intensity on FGG + force group was significantly higher than that on Force group, indicating that OPN expression in the alveolar bone with free gingival transplantation was higher than that in the Force group at 8 weeks. At 12 weeks, more active remodeling of the alveolar bone tissue on FGG + force group was still clearly observed, with obvious bone lacunae, and OPN expression was significantly higher than that on Force group. However, the overall expression of OPN at 12 weeks was weaker than that at 8 weeks. In Sham + force group, more active alveolar bone remodeling was also observed at 4 and 8 weeks, which decreased at 12 weeks. Moreover, there was no significant difference in the expression of bilateral OPN in Sham + force group at 4, 8, and 12 weeks. Bar charts more intuitively show the trend of OPN expression in FGG + force group (Figure 5C) and Sham + force group (Figure 5D) after different durations of force application, with the most significant difference between FGG + force and Force group of 8 weeks.

**FIGURE 6:**
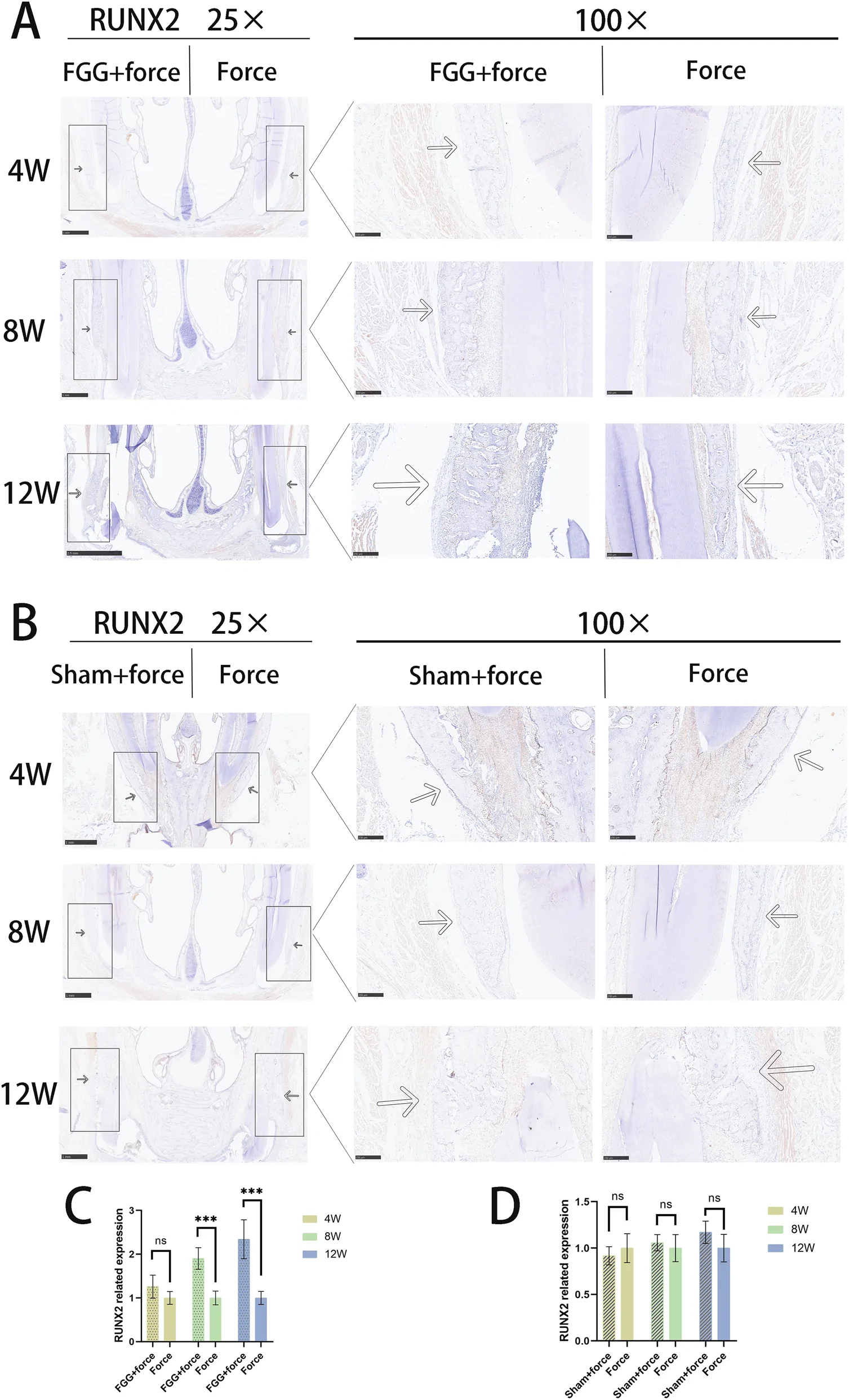
**(A)** The expression of RUNX2 in the bone tissue of the FGG + force group was higher than that of the force group, and it became more pronounced over time. **(B)** In the sham + force operation group, there were no significant differences in the expression of bilateral RUNX2 in bone tissue and periodontal ligament at 4 weeks, 8 weeks and 12 weeks. **(C,D)** The positive degree of immunohistochemical results of OPN and RUNX2 was analyzed by ImageJ software and represented by bar charts. (The arrow points to the location for observation and analysis.) (*p < 0.05, **p < 0.01, ***p < 0.001).

Immunohistochemical analysis of RUNX2 (Figure 6A) revealed progressive expression dynamics. At 4 weeks, FGG + force group demonstrated RUNX2 modestly elevated staining intensity compared to Force group. This differential amplified substantially by 8 weeks, with FGG + force group showing markedly intensified expression particularly within bone matrices and adjacent periodontal ligament regions. The most pronounced expression occurred at 12 weeks on FGG + force group, coinciding histologically with active bone remodeling and prominent resorption lacunae. And it could also be clearly observed that the remodeling of alveolar bone tissue on the surgical force-applying side of rats was more active. Sham + force group maintained consistent bilateral symmetry in RUNX2 distribution across all timepoints without significant inter-side variations (Figure 6B). Quantification confirmed a difference between the two groups increasing over time, trend of RUNX2 expression in the FGG + force group peaking at 12 weeks (Figures 6C,D).

## 4 Discussion

This study established a rat model of excessive orthodontic tooth movement of maxillary incisors that recapitulates the clinical scenario of orthodontic treatment in patients with thin gingival and alveolar bone phenotypes. In orthodontic tooth movement research, various experimental animal models are widely used (Ren et al., 2003). Rats have human-like gingival histology, featuring analogous keratinized epithelium (OE), sulcular epithelium (OSE), and junctional epithelium (JE) (Kiliaridis et al., 1985; Ren et al., 2004; Xu and Yang, 2023; Listgarten, 1975; De Rouck et al., 2009; Tajima et al., 1992). The rat incisor region’s anatomical features—thin buccal bone plates and narrow keratinized gingiva—provide an ideal platform to simulate the biomechanical environment of clinical thin gingiva. Stem cells in the labial cervical loop of rodent incisor roots continuously generate enamel and mediate the physiological elongation of incisors along the longitudinal axis. Nevertheless, this process is not only perpendicular to the direction of orthodontic force applied in this study, but also anatomically distinct from the buccal alveolar bone region investigated herein (Ren et al., 2004; Singer et al., 2019; Li et al., 2019). Integrating the above evidence with the practical outcomes of our animal model establishment, the modeling protocol adopted in this study proves to be reliable and efficient.

A critical prerequisite for this model was the successful induction of alveolar bone dehiscence and fenestration. The biomechanical parameters of orthodontic force—namely its magnitude and direction—are fundamental determinants of the balance between bone resorption and formation during tooth movement (Jacob et al., 2024). Regarding force magnitude, forces of 20–40 g are generally considered optimal for orthodontic tooth movement of rat molars, whereas Chen et al. demonstrated that applying 80 g of force to rat molars induces more pronounced osteoclast activity and alveolar bone resorption (Zheng et al., 2024). The 80 g force used in the present study was confirmed by micro-CT and histology to cause root exposure and cortical bone defects in the incisors of rats in the Sham and control groups. The amount of tooth movement (approximately 7.5 mm over 12 weeks) exceeded the physiological limit of the alveolar bone housing, a condition that is highly analogous to clinical “excessive tooth movement” in orthodontic treatment where teeth are intentionally moved beyond their original alveolar boundaries. While movement beyond the cortical plate without adjunctive procedures inevitably leads to attachment loss (Brugnami et al., 2018). A recent study indicates that proclination of mandibular incisors leads to an increase in the distance from the cementoenamel junction to the marginal bone crest, and total bone thickness is reduced after proclination (Kalina et al., 2022). Therefore, the combination of an excessive orthodontic force and movement toward the thinner side of the rat alveolar bone reliably recapitulates the pathological phenomenon of alveolar bone defects induced by excessive orthodontic tooth movement.

FGG may exerts a protective effect on local alveolar bone. Micro-CT analysis revealed that in the FGG + force group, the left side exhibited significantly greater bone height, thickness, bone volume, and bone mineral density (at 4 weeks) compared with the right side. These differences were not observed in the Sham + force or Force groups, indicating that the beneficial effect is specifically attributable to the grafted tissue rather than to surgical manipulation or force application alone. Notably, the differences in the FGG + force group reached statistical significance at 4 and 12 weeks but not at 8 weeks for several parameters (e.g., bone height and thickness at 8 weeks showed *p* = 0.068). This transient lack of significance may be due to the smaller sample size at the 8-week time point (n = 4) or to a biological trough in the remodeling process, where osteoclastic activity temporarily outweighs osteogenesis. Nevertheless, the consistent pattern of numerical improvement and the significant differences at two of three time points support an overall osteoprotective effect. H&E staining showed that while the Force and Sham + force groups exhibited progressive cortical bone discontinuity, reduced alveolar crest height and penetrating bone defects, the FGG + force group displayed complete coverage of the alveolar bone by the transplanted palatal mucosa, with stable attachment between the subepithelial connective tissue and the bone surface. Newly formed epithelial pegs developed at the recipient site from 4 weeks post-operation and persisted through 12 weeks, indicating successful integration and long-term survival of the graft. Our previous clinical study by Zhang et al. investigated the effect of preventive FGG on alveolar bone parameters in orthodontic patients undergoing labial tooth movement, and its clinical findings are highly aligned with the micro-CT and histological results of our present rat model (Zhang et al., 2023).

Immunohistochemical analysis revealed time-dependent upregulation of the osteogenic transcription factor RUNX2 and the bone matrix protein OPN in FGG + force group compared with the Force group. RUNX2 expression progressively increased from 4 weeks to 12 weeks, peaking at the final time point, which explains the histological presence of Haversian canals and enhanced osseous integration. OPN expression was most pronounced at 8 weeks and declined by 12 weeks. This temporal divergence is consistent with the distinct biological roles of these two proteins: RUNX2 acts as an early-to-intermediate master regulator that commits mesenchymal cells to the osteoblast lineage, whereas OPN is a late-stage marker associated with matrix mineralization and bone remodeling. By contrast, the Sham + force and Force groups showed only modest and transient RUNX2 expression in the periodontal ligament, with no significant upregulation of OPN. These findings suggest that the grafted keratinized tissue protects the local alveolar bone, an effect not recapitulated by surgery alone. The increased expression of RUNX2 and OPN in FGG + force group coincided temporally with the improvement in alveolar bone morphological parameters, providing molecular correlate of the observed osteoprotective effect.

The precise mechanisms by which FGG exerts its protective effect on alveolar bone remain to be fully elucidated, but several possibilities can be considered based on the present data and existing literature. First, the grafted keratinized gingiva may act as a physical barrier that buffers the transmission of orthodontic forces to the underlying periosteum and cortical bone (Miao et al., 2022). The newly formed epithelial pegs and well-integrated connective tissue layer observed in our histological sections are consistent with this barrier function. By distributing and attenuating mechanical stress, the graft could reduce local bone strain and thus limit the extent of force-induced bone resorption.

Second, surgical preparation of the recipient site may create a localized injury that activates resident progenitor cells. As reported, mucogingival surgery involving periosteal manipulation can release osteoprogenitors from the periosteum-alveolar bone interface, which then proliferate and contribute to new bone formation. In the present study, the periosteum was deliberately preserved during recipient site preparation, but the surgical incision and blunt dissection still inevitably disrupt the periosteal integrity to some extent, potentially triggering this progenitor-mediated osteogenic response.

Third, the graft itself may serve as a source of mesenchymal stem cells or osteogenic factors. Recent studies have revealed that, compared to non-keratinized vestibular mucosa, human palatal keratinized gingiva contains a significantly enriched population of gingival mesenchymal stem cells, 78% of these cells are localized in the lamina propria of the palatal keratinized tissue (Pink et al., 2023). These graft-derived cells may directly differentiate into osteoblasts or, more likely, secrete paracrine factors that recruit and activate host osteoprogenitors. Additionally, the grafted tissue may modulate the local immune microenvironment in a pro-regenerative direction, as immune-stromal cell interactions have been shown to be central regulators of bone repair (Xiong et al., 2022).

This research also has limitations. There are physiological differences between rodent maxillary incisors and human permanent dentition, as well as discrepancies in the anatomical structure of the periodontal ligament. The existing control settings cannot completely eliminate the confounding effects of surgical manipulation, systemic factors, and the force application device itself on the experimental results, which may compromise the extrapolation of the findings related to orthodontic biomechanics and alveolar bone remodeling in this study to human clinical scenarios. Additional core molecules regulating osteogenic and osteoclastic processes, including TRAP, RANKL, BMP-2, and ALP, were not detected in this study, making it impossible to comprehensively characterize the bidirectional regulatory balance between osteogenesis and osteoclastogenesis during alveolar bone remodeling. The specific molecular regulatory mechanism underlying the osteoprotective effect of free gingival graft and its long-term clinical application value still need to be clarified by subsequent more in-depth studies with extended observation periods. Another limitation of this study is the relatively small sample size per time point. Despite the modest sample size, the paired Wilcoxon tests revealed consistently significant differences in the FGG + force group at 4 and 12 weeks (*p* < 0.05). The results should be interpreted conservatively, and future studies with larger sample sizes are warranted to confirm the observed effect sizes.

## 5 Conclusion

In this study, we established an excessive orthodontic tooth movement model of maxillary anterior teeth in Sprague-Dawley rats to simulate the pathological changes of alveolar bone resorption and bone defects that frequently occur during orthodontic treatment in patients with thin gingiva and thin alveolar bone. Using micro-CT and histopathological examination, we found that performing free gingival grafting (FGG) prior to orthodontic intervention alleviated orthodontic force-induced alveolar bone resorption during excessive tooth movement. This study provides a referable rat model for exploring the regulatory effect of keratinized gingival grafting on alveolar bone remodeling, and lays a preliminary experimental foundation for the prophylactic application of FGG in orthodontic patients with a thin gingiva-thin bone phenotype.

## Data Availability

The raw data supporting the conclusions of this article will be made available by the authors, without undue reservation.

## Supplementary material

SUPPLEMENTARY FIGURE S1
**(A)** The mucosal donor site on the rat's palate had fully recovered 10 days postoperatively, with no impact on the rats' feeding. **(B)** Masson staining image of keratinized gingival mucosa in the upper palate of rats. **(C)** The device is compressed upon installation and exerts equal and opposite forces (black arrows). **(D)** Intensity of force was measured using a digital force gauge (ZP-5N, Shenzhen AILIGU Instrument Co., Ltd, China). **(E)** The device was compressed to a pre-determined compression length, and the peak force value was recorded by a computer. **(F)** The measured data were analyzed and fitted; the fitting results conformed to Hooke’s law.

SUPPLEMENTARY FIGURE S2
**(A)** Immunohistochemical labeling of OPN. In the blank group of rats without force application, the buccal bones of the bilateral anterior teeth were thin, and the osteogenic and osteoclastic activities of the alveolar bone were inactive. No obvious positive staining was observed at 4 weeks and 8 weeks. **(B)** RUNX2 immunohistochemical staining showed that a small amount of positive staining was observed near the periodontal ligament close to the tooth surface in the 4-week sample, but this positive staining disappeared after 8 weeks, and no significant expression difference was observed on the left and right sides. **(C,D)** The positive degree of immunohistochemical results of OPN and RUNX2 was analyzed by ImageJ software and represented by bar charts (The arrow points to the location for observation and analysis).
